# The GAIN Registry — a New Prospective Study for Patients with Multi-organ Autoimmunity and Autoinflammation

**DOI:** 10.1007/s10875-023-01472-0

**Published:** 2023-04-21

**Authors:** Paulina Staus, Stephan Rusch, Sabine El-Helou, Gabriele Müller, Máté Krausz, Ulf Geisen, Andrés Caballero-Oteyza, Renate Krüger, Shahrzad Bakhtiar, Min Ae Lee-Kirsch, Maria Fasshauer, Ulrich Baumann, Bimba Franziska Hoyer, João Farela Neves, Michael Borte, Maria Carrabba, Fabian Hauck, Stephan Ehl, Peter Bader, Horst von Bernuth, Faranaz Atschekzei, Mikko R. J. Seppänen, Klaus Warnatz, Alexandra Nieters, Gerhard Kindle, Bodo Grimbacher

**Affiliations:** 1 Division Methods in Clinical Epidemiology, Institute of Medical Biometry and Statistics, Faculty of Medicine and Medical Center, University of Freiburg, Freiburg, Germany; 2 Institute for Immunodeficiency, Center for Chronic Immunodeficiency (CCI), Faculty of Medicine, Medical Center, University of Freiburg, Breisacher Str. 115, 79106 Freiburg, Germany; 3 Department of Rheumatology and Immunology, Cluster of Excellence RESIST (EXC 2155), Hannover Medical School, Hanover, Germany; 4 Department of Rheumatology and Clinical Immunology, Medical Center - University of Freiburg, Faculty of Medicine, University of Freiburg, Freiburg, Germany; 5 Faculty of Biology, Albert-Ludwigs-University of Freiburg, Freiburg, Germany; 6 Excellence Center for Inflammation Medicine, Clinic for Rheumatology and Clinical Immunology, University Hospital Schleswig-Holstein, Campus Kiel, Kiel, Germany; 7 Department of Pediatric Respiratory Medicine, Immunology and Critical Care Medicine, Charité - Universitätsmedizin Berlin, Berlin, Germany; 8 Division for Stem Cell Transplantation, Immunology and Intensive Care Medicine, Hospital for Children and Adolescents, University Hospital, Goethe University, Frankfurt am Main, Germany; 9 Department of Pediatrics, University Hospital and Medical Faculty Carl Gustav-Carus, Technische Universität Dresden, Dresden, Germany; 10 Hospital for Children & Adolescents, St. Georg Hospital, Leipzig, Germany; 11 Academic Teaching Hospital of the University of Leipzig, Immunodeficiency Center Leipzig (IDCL), Leipzig, Germany; 12 Department of Paediatric Pulmonology, Allergy and Neonatology, Hannover Medical School, Hanover, Germany; 13 Primary Immunodeficiencies Unit, Hospital Dona Estefânia, Centro Hospitalar de Lisboa Central, EPE, Lisbon, Portugal; 14 CHRC, Comprehensive Health Research Centre, NOVA Medical School, NOVA University of Lisbon, Lisbon, Portugal; 15 CEDOC, Chronic Diseases Research Center, NOVA Medical School, NOVA University of Lisbon, Lisbon, Portugal; 16 Dipartimento di Medicina Interna, Fondazione IRCCS Ca’ Granda Ospedale Maggiore Policlinico, UOS Malattie Rare, Milan, Italy; 17 Department of Pediatrics, Dr von Hauner Children’s Hospital, University Hospital, Ludwig Maximilians Universität München, Munich, Germany; 18 Berlin Institute of Health at Charité – Universitätsmedizin Berlin, Berlin, Germany; 19 Department of Immunology, Labor Berlin GmbH, Berlin, Germany; 20 Charité - Universitätsmedizin Berlin, corporate member of Freie Universität Berlin, Humboldt-Universität zu Berlin, and Berlin Institute of Health (BIH), Berlin-Brandenburg Center for Regenerative Therapies (BCRT), Berlin, Germany; 21 The Rare Disease and Pediatric Research Centers, Hospital for Children and Adolescents and Adult Immunodeficiency Unit, Inflammation Center, University of Helsinki and HUS Helsinki, University Hospital, Helsinki, Finland; 22 DZIF - German Center for Infection Research, Satellite Center Freiburg, Freiburg, Germany; 23 CIBSS – Centre for Integrative Biological Signalling Studies, Albert-Ludwigs University, Freiburg, Germany; 24 RESIST - Cluster of Excellence 2155 to Hanover Medical School, Satellite Center Freiburg, Freiburg, Germany

**Keywords:** Inborn error of immunity, Autoimmunity, Autoinflammation, Immune-dysregulation, Primary immunodeficiency, Epidemiology, Rare diseases

## Abstract

Patient registries are a very important and essential tool for investigating rare diseases, as most physicians only see a limited number of cases during their career. Diseases of multi-organ autoimmunity and autoinflammation are especially challenging, as they are characterized by diverse clinical phenotypes and highly variable expressivity. The GAIN consortium (German multi-organ Auto Immunity Network) developed a dataset addressing these challenges. ICD-11, HPO, and ATC codes were incorporated to document various clinical manifestations and medications with a defined terminology. The GAIN dataset comprises detailed information on genetics, phenotypes, medication, and laboratory values. Between November 2019 and July 2022, twelve centers from Europe have registered 419 patients with multi-organ autoimmunity or autoinflammation. The median age at onset of symptoms was 13 years (IQR 3–28) and the median delay from onset to diagnosis was 5 years (IQR 1–14). Of 354 (84.5%) patients who were genetically tested, 248 (59.2%) had a defined monogenetic cause. For 87 (20.8%) patients, no mutation was found and for 19 (4.5%), the result was pending. The most common gene affected was *NFkB1* (48, 11.5%), and the second common was *CTLA4* (40, 9.5%), both genetic patient groups being fostered by specific research projects within GAIN. The GAIN registry may serve as a valuable resource for research in the inborn error of immunity community by providing a platform for etiological and diagnostic research projects, as well as observational trials on treatment options.

**Supplementary Information:**

The online version contains supplementary material available at 10.1007/s10875-023-01472-0.

## Introduction

The management of patients with rare diseases is challenging for treating physicians, as many questions of patients stay unanswered due to the lack of data and the sparse knowledge for making treatment decisions. In addition, research to address these questions is hindered by the very limited availability of research samples of these rare patients. Patients with immune dysregulation and multi-organ autoimmunity belong to a group of patients with diverse rare genetic causes presenting with partly life-threatening autoimmune or autoinflammatory diseases of several organs. We still do not know how prevalent these diseases are, and detailed clinical, genetic, and quality of life information from these patients is lacking. Our aim was to document this type of data in a structured and regular manner and share it with the research community. Before, there was no data structure which was capable of capturing the diverse phenotypes and genotypes of patients with multi-organ autoimmune or autoinflammatory diseases in sufficient detail. Here, we present the GAIN registry, capable of addressing these challenges using international classification systems. This registry was set up in the context of the GAIN consortium (German multi-organ AutoImmunity Network; www.g-a-i-n.de). The registry is its central project and interconnects all other research projects within the consortium. We integrated the GAIN registry on the online registry platform of the ESID registry (European Society for Immunodeficiency; https://esid.org/Working-Parties/Registry-Working-Party/ESID-Registry). The ESID registry is a well-established, European-wide registry in the field of primary immunodeficiencies. Every documenting center of the ESID registry can easily participate in the GAIN registry project. The main research question addresses the prevalence of patients with multi-organ autoimmunity or autoinflammation. Second, we aim at answering basic questions of patients such as, “What can I expect from life in the future?” To answer this, we collect whenever possible the timing of events to document the disease progression. Third, our detailed dataset aims to address the relationship between the underlying gene variant and the actual expressivity of the disease. In addition, we designed the registry to serve as a platform for more detailed research projects. Patients with specific genetic or phenotypic traits can be identified. Detailed information on infections and the causing pathogens, organ involvements, comorbidities, allergies, vaccination status, treatment regiments, and genetic information may be documented into the registry.

## Methods

### Organization

The GAIN registry is part of the German multi-organ AutoImmunity Network (GAIN) consortium (BMBF, funding code 01GM1910A) and complements wet-lab research projects, a clinical pharmacological trial (ABACHAI), and a joint consortial GAIN Biobank, all part of the first GAIN funding period from 2019 to 2022.

### How to Participate

The GAIN registry was implemented on the platform of the European Society for Immunodeficiency (ESID) registry [1] and is hence open to all centers that wish to participate and conclude an agreement with the ESID registry and have the approval of their local ethics committees. The ESID registry includes a core dataset (level 1), which is also always documented for GAIN patients. The detailed GAIN dataset is therefore an ESID level 2 dataset. All participating centers can hand in research proposals to the GAIN consortium. Patients with written informed consent of the ESID registry may be included into the GAIN registry. No other patient consent is necessary. Identifying information of patients are not stored in the ESID database, but can be matched locally [2] depending on the users’ access rights. For more details and information of the technical background of the ESID registry, see Scheible et al. (2019) [1]. All GAIN patients are directly made available to the ESID registry for other secondary research level 2 studies.

### Research Goals

First, the level 2 GAIN registry dataset in combination with the core ESID registry dataset (level 1) was designed to estimate the minimal prevalence and key parameters and characteristics of immune dysregulatory diseases as outlined in Table 1. Only the minimal prevalence can be estimated as the participation in the registry is voluntary.

**Table 1:** Research interest of the GAIN registry

1	Minimal prevalence of immune-dysregulatory diseases
2	Median age of disease onset
3	Median age at diagnosis and diagnostic delay
4	Median survival
5	Detailed description of phenotypic manifestations of the disease, including infections and comorbidities
6	Detailed description of treatment
7	Detailed description of genetic variants
8	Quality of life of patients

Second, we want to investigate — by longitudinal follow-up — risk factors associated with the expressivity of the disease, especially disease initiation, progression, and treatment response. With detailed description of genetic variants, we hope to identify new genetic and molecular pathways associated with multi-organ autoimmunity/autoinflammation.

Third, our detailed dataset aims to address the relationship between the underlying gene variant and the actual expressivity of the disease. We want to offer a platform for more detailed research projects for various subsets of multi-organ autoimmune/autoinflammatory patient groups, which alone would never have the resources to build up their own registry.

### Inclusion Criteria for Patients

Patients are eligible for documentation into the GAIN registry, when they show a multi-organ pathology with either an autoimmunity or an autoinflammation phenotype or have a proven pathogenic mutation in a gene known to cause multi-organ autoimmunity/autoinflammation.

A list of genes known to cause multi-organ autoimmunity/autoinflammation can be found in Table 2. Genes are being added to the list whenever new evidence emerges. However, we also include patients into the GAIN registry with an unknown genetic cause to be able to investigate unknown genetic determinants. In addition, patients need to fulfill the ESID registry criteria [3]. The treating physician decides whether their patients fulfill these inclusion criteria. Using these criteria, we aim at including patients in the longitudinal data collection who are carriers of a mutation but do not show a multi-organ autoimmune/autoinflammation phenotype yet. Patients with an acquired immunodeficiency are not included into the study.

**Table 2:** Candidate genes known to cause multi-organ autoimmunity or autoinflammation

Gene abbreviation	Gene name
*AIRE*	Autoimmune regulator
*ADA2*	Adenosine deaminase 2
*BACH2*	BTB domain and CNC homolog 2
*CD25/IL2-RA*	Interleukin 2 receptor subunit alpha
*CTLA4*	Cytotoxic T-lymphocyte associated protein 4
*FOXP3*	Forkhead box P3
*ICOS*	Inducible T cell costimulator
*LAT*	Linker for activation of T cells
*LRBA*	LPS responsive beige-like anchor protein
*NFKB1*	Nuclear factor kappa B subunit 1
*NFKB2*	Nuclear factor kappa B subunit 2
*PIK3CD*	Phosphoinositide 3 kinase catalytic subunit delta
*PIK3R1*	Phosphoinositide 3 kinase regulatory subunit 1
*PRKCD*	Protein kinase C delta
*RELA*	Nuclear factor kappa B subunit 3
*SOCS1*	Suppressor of cytokine signaling 1
*STAT1-GOF*	Signal transducer and activator of transcription 1
*STAT3-GOF*	Signal transducer and activator of transcription 3
*TNFRSF13B*	Tumor necrosis factor receptor superfamily member 13B
*TPP2*	Tripeptidyl peptidase 2

*GOF*, gain-of-function

### Ongoing Projects and Their Research Goals

The detailed data structure of the registry offers to answer specific research questions in subgroups of patients. Ongoing projects within the registry are listed in Table 3 with their inclusion criteria and research goals. Most projects use the possibility to capture detailed information on the history of symptoms, organ manifestations, and infections for the patients to describe their clinical phenotype. Three projects use the detailed documentation of treatment such as the history of prescribed drugs with its dose, frequency, treatment duration, adverse events, and outcome for the patient. Other possible research questions which may be answered by the dataset are as follows:

- How are titer responses to vaccinations related to the infection history?
- Do certain patient subgroups suffer more often from allergies?
- Which type of cancer is more prevalent in patients with a given genetic diagnosis?
- Are these cancers associated with the persistence of specific viruses?

**Table 3:** Ongoing projects within the GAIN registry

Project name	CTLA4 insufficiency	NFKB1 insufficiency	NFKB2 insufficiency	ADA2 deficiency	CARD11-GOF disease	RELA insufficiency	STAT3-GOF disease
Responsible person	Bodo Grimbacher	Bodo Grimbacher	Bodo Grimbacher	Fabian Hauck	Fabian Hauck	Min-Ae Lee-Kirsch	Stephan Ehl
Inclusion criteria	All patients with pathogenic mutations in *CTLA4*	All patients with pathogenic mutations in *NFKB1*	All patients with pathogenic mutations in *NFKB2*	All patients with pathogenic mutations in *ADA2*	All patients with pathogenic gain-of-function mutations in *CARD11*	All patients with pathogenic mutations in *RELA*	All patients with pathogenic gain-of-function mutations in *STAT3*
Exclusion criteria	Not consented individuals	Not consented individuals	Not consented individuals	Not consented individuals	Not consented individuals	Not consented individuals	Not consented individuals
No. of patients	40	48	18	10	starting 01/2023	starting 01/2023	12
Main research goals	Which complications are common in CTLA4 insufficiency? Which drugs are successfully used to treat these?	Which complications are common in NFKB1 insufficiency? Which drugs are successfully used to treat these?	Which complications are common in NFKB2 insufficiency? Which drugs are successfully used to treat these?	Describe the extended clinical phenotype of patients with biallelic mutations in *ADA2*	Describe the extended clinical phenotype of patients with gain-of-function mutations in *CARD11*	Describe the extended clinical phenotype of patients with mutations in *RELA*	Describe the extended clinical phenotype of patients with gain-of-function mutations in *STAT3*
Secondary research goals	Identify biomarkers for treatment response and treatment failure	Identify biomarkers for treatment response and treatment failure	Identify biomarkers for treatment response and treatment failure. Assess the importance of anti-interferon antibodies in patients with NFKB2 insufficiency	Identify biomarkers for treatment response and treatment failure	Identify biomarkers for treatment response and treatment failure	Identify biomarkers for treatment response and treatment failure	Identify biomarkers for treatment response and treatment failure
Starting time	2019	2019	2019	2019	2023	2023	2019
Planned project duration	9 years	9 years	9 years	9 years	6 years	6 years	9 years

### Recruiting Measures

Currently, twelve centers are documenting into the GAIN registry, ten originating from various regions in Germany and one center in Italy and one in Portugal each (Supplementary Table 1). Any ESID documenting center may include GAIN patients prospectively (patient has not previously been entered into the ESID registry) or retrospectively (patient had been documented into ESID before and now the GAIN level 2 dataset is being filled). Moreover, with local ethics committee approval, deceased patients may also be included.

### Data Sources

Information entered into the GAIN registry derives mainly from three sources: The first and most important are (electronic) patient records. Information needs to be manually retrieved from medical letters and reports, coded if necessary, and entered into the online forms of the registry. Second are local research databases, for example, with genetic information on patients. The third data sources are electronic and paper questionnaires, designed to support the collection of information, which is not regularly available from patient records, such as quality of life data. For quality assurance, the documentation specialists will check documented data for plausibility and completeness and resolve open issues with the treating physicians.

### Statistics

The analyses of the collected dataset focused on simple descriptive summary statistics, performed with R version 4.2.2 and Microsoft Excel 2010. If exact dates were missing but necessary for calculation, the middle of the month (15th) or the middle of the year (1st of July) was assumed. For the summary of genes with genetic variants, information from the GAIN registry was supplemented with information from ESID level 1 entries.

## Results

### Design and Implementation of the GAIN Registry

**Fig. 1:**
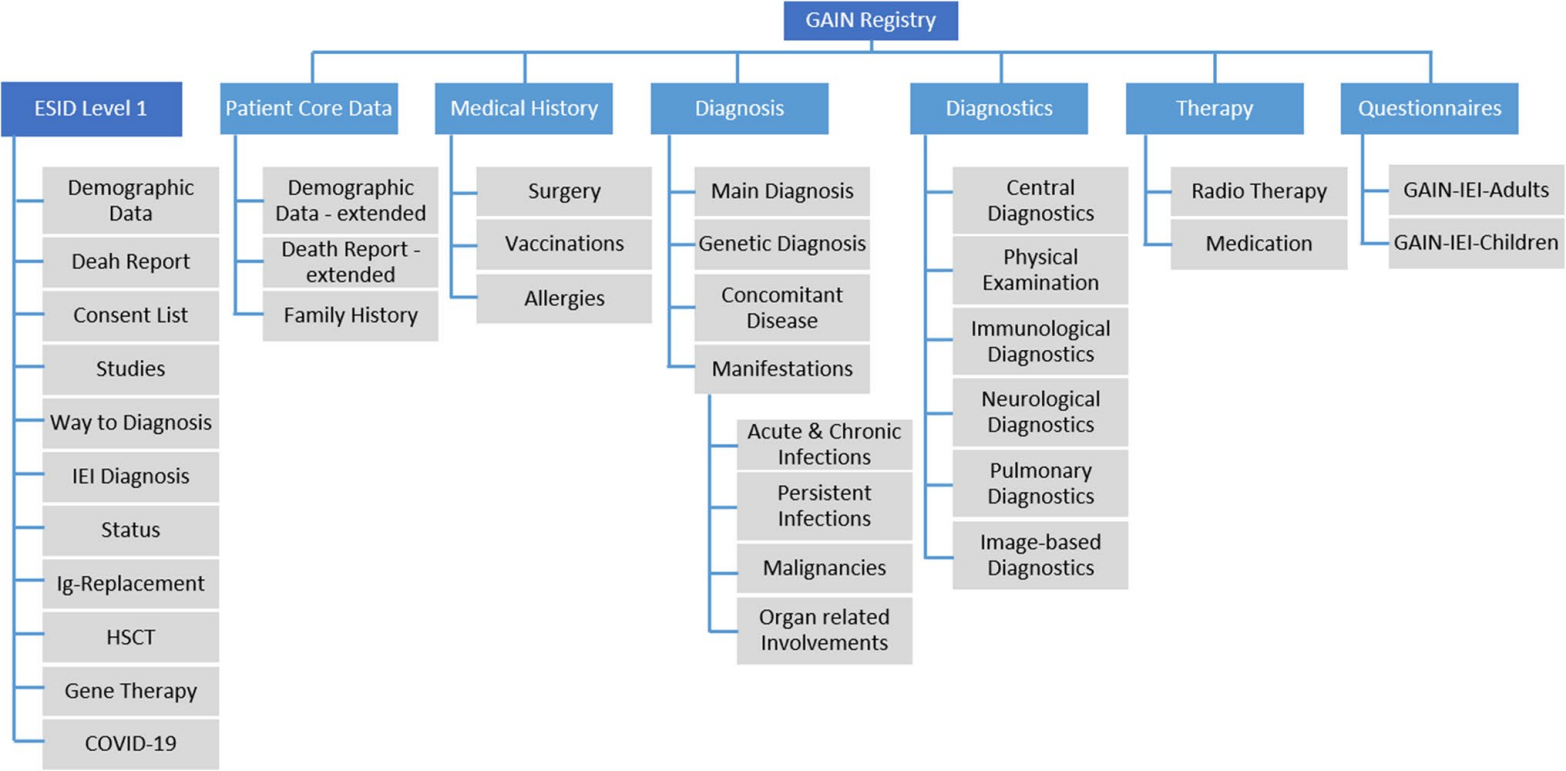
Overview of GAIN registry dataset structure and the ESID basic level 1 dataset

To overcome the need and expenses of creating a new registry for each new rare disease subgroup, we designed and implemented a dataset which is capable of capturing various manifestations of different forms of autoimmune/autoinflammatory diseases. In order to mirror a complex disease, a data structure with a complexity similar to an electronic patient record was developed (Fig. 1). The full GAIN entry forms can be accessed by the demonstration version of the ESID registry via https://cci-esid-reg-demo-app.uniklinik-freiburg.de/EERS with the username “demouser” and the password “Demo-2019” [1]. The GAIN dataset is designed to collect prospective information. Information which does not change, such as information on consanguinity, is only collected once. Information on laboratory and diagnostic values, as well as questionnaires, can be documented at each visit. A visit and documentation into the GAIN registry is aimed for at least once a year. In addition, therapies, infections, malignancies, and other organ-related symptoms and pathologies are captured in lists, including start and end dates. A GAIN-specific user guide that amends the general ESID registry user manual and answers frequently asked questions (in English) and a video tutorial (in German) were developed to support documentation and are available at the GAIN project website https://www.g-a-i-n.de/register/. From November 2019 on, patients have been registered into the GAIN registry and basic information was entered into the ESID level 1 dataset. Since the beginning of the year 2021, the GAIN dataset level 2 was also available for documentation.

### Coding Systems for Increased Interoperability

Standardized terminology is a prerequisite to merge or to compare datasets. To foster the interoperability and sustainability of the GAIN dataset, it is based on international coding systems. The main clinical diagnosis of the patient is coded with the ORPHA nomenclature and ICD (International Classification of Diseases) codes [4]. The ORPHA nomenclature is maintained by the European Commission funded consortium “orphanet” and was developed especially for rare diseases [5].

The ICD codes are the global standard for diagnostic health information, developed and maintained by the WHO. We implemented the ICD-11 version, the more detailed and updated version of the commonly used ICD-10 code, as it was meant to replace ICD-10 in clinical information systems in the medium term. ICD-11 codes are being used to code all disease manifestations of immunodeficient patients, including infections, malignancies, and organ-related manifestations of autoimmunity or autoinflammation. In addition, the extension code of ICD-11 is used for the classification of pathogens, allergens, body sites, and tissues in the GAIN registry.

For the documentation of symptoms, we opted for the Human Phenotype Ontology (HPO) and implemented it as a REST-Application Programming Interface in the registry. This type of implementation assures that coding requests are sent directly to HPO servers; thereby, the evolving ontology is always up to date. The HPO is an international ontology, developed by the Monarch initiative, funded by the National Institute of Health of the USA [6].

Medications are coded by their ingredients using the ATC code (anatomical-therapeutic-chemical classification) [7]. In addition, if available, the full product name of the medication is documented based on a table requested from the European Medicines agency, comprising all drugs approved in Europe [8]. The documentation of drug allergies also uses this set of codes.

Classifications are performed by the treating physician or the documentation specialist. To document the classification process, also the original wording of the primary data source is recorded in original language.

### Preselection of Common Diseases and Symptoms

Classification systems and ontologies enable a specific documentation of a large repertoire of disease and symptom entities. To facilitate coding, we included suggestions for common malignancies, organ involvements, and symptoms for patients with multi-organ autoimmunity/autoinflammation. These suggestions were developed by experienced clinicians of the GAIN consortium. When one suggestion is chosen, the corresponding ICD-11 or HPO code is complemented automatically.

### Genetic Diagnosis

To enable phenotype-genotype correlations, the genetic information needs to be documented in detail. When collecting genetic data from different laboratories and different genetic tests, a common language and the specification of the reference genome are needed; otherwise, information might become ambiguous. To ensure standardized data entry of the genetic variant, users are asked to verify the HGVS (human genome variation society) conform format with the syntax checker developed by Lefter et al. [9]. In addition, the transcript ID can be selected either in Ensemble or RefSeq by searching the gene name to avoid typing errors [10, 11].

### Capturing Incomplete Data

A good data quality of primary sources is a prerequisite for good data quality in the registry. Information is available in varying degrees of completeness in primary data sources (medical letters and records) and consequently in the GAIN registry. The GAIN dataset allows data entry of partly available information to use as much information as possible. If the exact date for an event is unknown, only the year or the year and the month can be specified. In addition, we offer the options, “Currently unknown,” “Truly unknown,” “Currently ongoing,” “Currently unknown if ongoing,” “Not ongoing but date currently unknown,” and “Not ongoing but date truly unknown” for diseases and symptoms to take into account the prospective nature of the documentation.

### Cohort Description

Until the 27th of July 2022, 419 patients have been registered. 38.7% (162) of all patients were prospectively included, and 61.3% (257) retrospectively, as they had been already registered into the ESID registry. For 64.2% (269) of patients, the basic ESID level 1 dataset and two follow-ups or more had been documented. For 45.4% (132), the detailed GAIN dataset has been documented at least once. A total of 354 patients (86.5%) were genetically tested. For 59.2% (248), at least one gene variant was reported. In total, variants in 39 different genes were reported. Most registered patients had a defect in the *NFKB1* gene (48 patients, 11.5%; see Table 4).

**Table 4:** Characteristics of patients in the GAIN registry

Characteristics	Total (*n* = 419)	No.	%
Gender	Male	201	48.0%
	Female	217	51.8%
	Missing	1	0.2%
Age^a^	Mean age (SD), 40 (±19)		
	0 – 9	25	6.0%
	10 – 19	49	11.7%
	20 – 29	67	16.0%
	30 – 39	69	16.5%
	40 – 49	61	14.6%
	50 –59	84	20.0%
	60 – 69	41	9.8%
	70 – 79	17	4.1%
	80+	6	1.4%
Status	Alive	399	95.2%
	Deceased	14	3.3%
	Discharged	1	0.2%
	Lost to follow-up	5	1.2%
Mutated gene^b^	*NFKB1*	48	11.5%
	*CTLA4*	40	9.5%
	*TNFRSF13B*	39	9.3%
	*NFKB2*	18	4.3%
	*LRBA*	13	3.1%
	*STAT3 GOF*	12	2.9%
	*ADA2*	10	2.4%
	*STAT1*	9	2.1%
	*STAT3*	9	2.1%
	*IKZF1*	7	1.7%
	*PIK3CD*	7	1.7%
	*ICOS*	6	1.4%
	*FAS*	5	1.2%
	*UNC13D*	3	0.7%
	*BACH2*	2	0.5%
	*CARMIL2*	2	0.5%
	*FLG*	2	0.5%
	*MST1*	2	0.5%
	*SH2D1A*	2	0.5%
	Other	22	5.3%
	Not tested	43	10.3%
	No mutation found	87	20.8%
	No information available^c^	41	9.8%

^a^In case of death, lost to follow-up, and discharge, age at death or last information was used

^b^Several mutated genes per patient are possible. Therefore, percentages do not sum up to 100%

^c^Pending result, or no information

We compared the gene variants of the GAIN registry with the main categories of the International Union of Immunological Societies (IUIS) tables [12], which categorized the genetic defects in different disease classes. Patients in the GAIN registry did not only fall into the “Table 4 Diseases of Immune Dysregulation” or “Table 7 Autoinflammatory Disorders” but also in more distant main categories as seen in Table 5. One patient inherited a gene variant in a gene not listed by the IUIS tables.

**Table:** Table 5

Table 1 Immunodeficiencies affecting cellular and humoral immunity	
*DCLRE1C (ARTEMIS)*	DNA Cross-Link Repair 1C
*ICOS*	Inducible T Cell Costimulator
*IKBKB*	Inhibitor Of Nuclear Factor Kappa B Kinase Subunit Beta
*IKZF1*	IKAROS Family Zinc Finger 1
*STK4 (MST1)*	Serine/Threonine Kinase 4
Table 2 Combined immunodeficiencies with associated or syndromic features	
*IKBKB*	Inhibitor Of Nuclear Factor Kappa B Kinase Subunit Beta
*SPINK5*	Serine Peptidase Inhibitor Kazal Type 5
*STAT3*	Signal Transducer and Activator Of Transcription 3
*WAS*	WASP Actin Nucleation Promoting Factor
Table 3 Predominantly Antibody Deficiencies	
*BTK*	Bruton Tyrosine Kinase
*IKZF1*	IKAROS Family Zinc Finger 1
*NFκB1*	Nuclear Factor Kappa B Subunit 1
*NFκB2*	Nuclear Factor Kappa B Subunit 2
*PIK3CD*	Phosphoinositide 3 Kinase Catalytic Subunit Delta
*PIK3R1*	Phosphoinositide 3 Kinase Regulatory Subunit 1
*TNFRSF13B*	Tumor Necrosis Factor Receptor Superfamily Member 13B
*TNFRSF13C*	TNF Receptor Superfamily Member 13C
*UNG*	Uracil DNA Glycosylase
Table 4 Diseases of Immune Dysregulation	
*AIRE*	Autoimmune Regulator
*BACH2*	BTB Domain and CNC Homolog 2
*CARMIL2*	Capping Protein Regulator And Myosin 1 Linker 2
*CD70*	CD70 Molecule
*CTLA4*	Cytotoxic T-Lymphocyte Associated Protein 4
*FAS*	Fas Cell Surface Death Receptor
*FOXP3*	Forkhead Box P3
*LRBA*	LPS Responsive Beige-Like Anchor Protein
*RIPK1*	Receptor Interacting Serine/Threonine Kinase 1
*SH2D1A*	SH2 Domain Containing 1A
*SOCS1*	Suppressor of Cytokine Signalling 1
*STAT3*	Signal Transducer and Activator Of Transcription 3
*UNC13D*	Unc-13 Homolog D
*XIAP*	X-Linked Inhibitor Of Apoptosis
Table 5 Congenital defects of phagocyte number or function	
*CYBC1*	Cytochrome B-245 Chaperone 1
*JAGN1*	Jagunal Homolog 1
*WAS*	WASP Actin Nucleation Promoting Factor
Table 6 Defects in intrinsic and innate immunity	
*STAT1*	Signal Transducer and Activator Of Transcription 1
Table 7 Autoinflammatory Disorders	
*ADA2*	Adenosine Deaminase 2
*NLRP3*	NLR Family Pyrin Domain Containing 3
*PLCG2*	Phospholipase C Gamma 2
*SYK*	Spleen Associated Tyrosine Kinase
*TNFAIP3*	TNF Alpha Induced Protein 3
*TNFRSF1A*	TNF Receptor Superfamily Member 1A
*No IUIS Main category table*	
*FLG*	Filaggrin

#### The Patient’s Path to Diagnosis

**Fig. 2:**
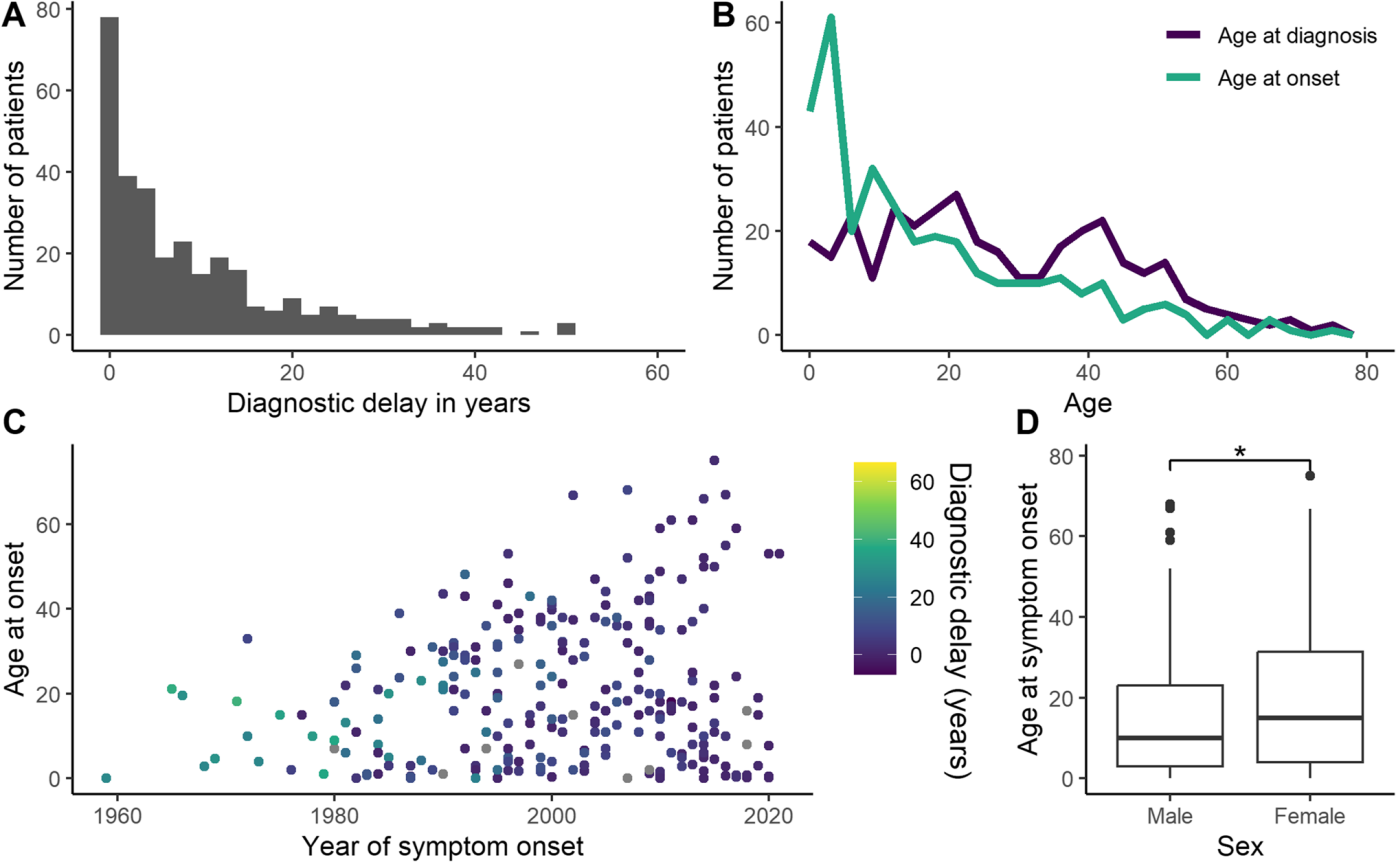
Delayed diagnosis for patients with multi-organ autoimmunity/autoinflammation. **A** Histogram of diagnostic delay. **B** Frequency polygon for age at diagnosis and age at onset in years, 3 years grouped for plotting. **C** Age at onset and diagnostic delay for the calendar year of symptom onset. It is difficult to estimate calendar time effects with our cohort. Patients with symptom onset several decades ago and in later age are underrepresented in the cohort, due to the prospective inclusion of mostly living patients in the registry. **D** Male patients experience symptom onset earlier than females. Wilcoxon rank sum test *p* = 0.024, *n* = 352

In any healthcare system, it typically takes a long time to diagnose patients with rare diseases. The resulting delay of an appropriate treatment leads to increased patient morbidity and considerable consumption of healthcare resources [13, 14]. As our patient group suffers from a wide range of symptoms, we were interested in the time span from symptom onset to clinical diagnosis, i.e., the diagnostic delay. Symptom onset was defined as first symptom suggestive of an inborn error of immunity (IEI) based on the retrospective physicians’ assessment. The diagnostic delay spans a large range (see Fig. 2A). After presenting with symptoms suggestive of IEI, 22.9% (88) of patients were diagnosed within 1 year. However, the median time of diagnostic delay was 5 years (IQR 1–14). A large proportion of patients lacked a clear diagnosis after symptom onset for several years and even decades.

Options for documenting first symptoms were infection, immune dysregulation, malignancy, syndromal manifestations, other, and unknown. For most patients, clinical symptoms suggested an IEI (385 patients, 91.9%). No symptom was apparent for 14 patients (3.3%) who were diagnosed by laboratory values (10 patients) or because of a known familial genetic defect (four patients). For 20 patients (4.8%), this information was unknown. Of the 385 patients with IEI-related symptoms, infection (285 patients, 74.0%) was the most prominent, followed by immune dysregulation (196, 50.9%). Immune dysregulation was defined as lymphoproliferation (splenomegaly, hepatomegaly, lymphadenopathy), granuloma formation, autoimmunity (e.g., cytopenia, thyroid disease, joint disease, hepatitis, vitiligo, alopecia, diabetes), inflammatory bowel disease, celiac disease, vasculitis, eczema, or autoinflammatory disease. Both infections and symptoms of immune dysregulation were apparent for 119 patients (28.4%) who were diagnosed based on symptoms. Less frequent as presenting symptom was a syndromal phenotype (18, 4.7%), malignancies (6, 1.6%), and others with unclear categorization (24, 6.2%).

**Fig. 3:**
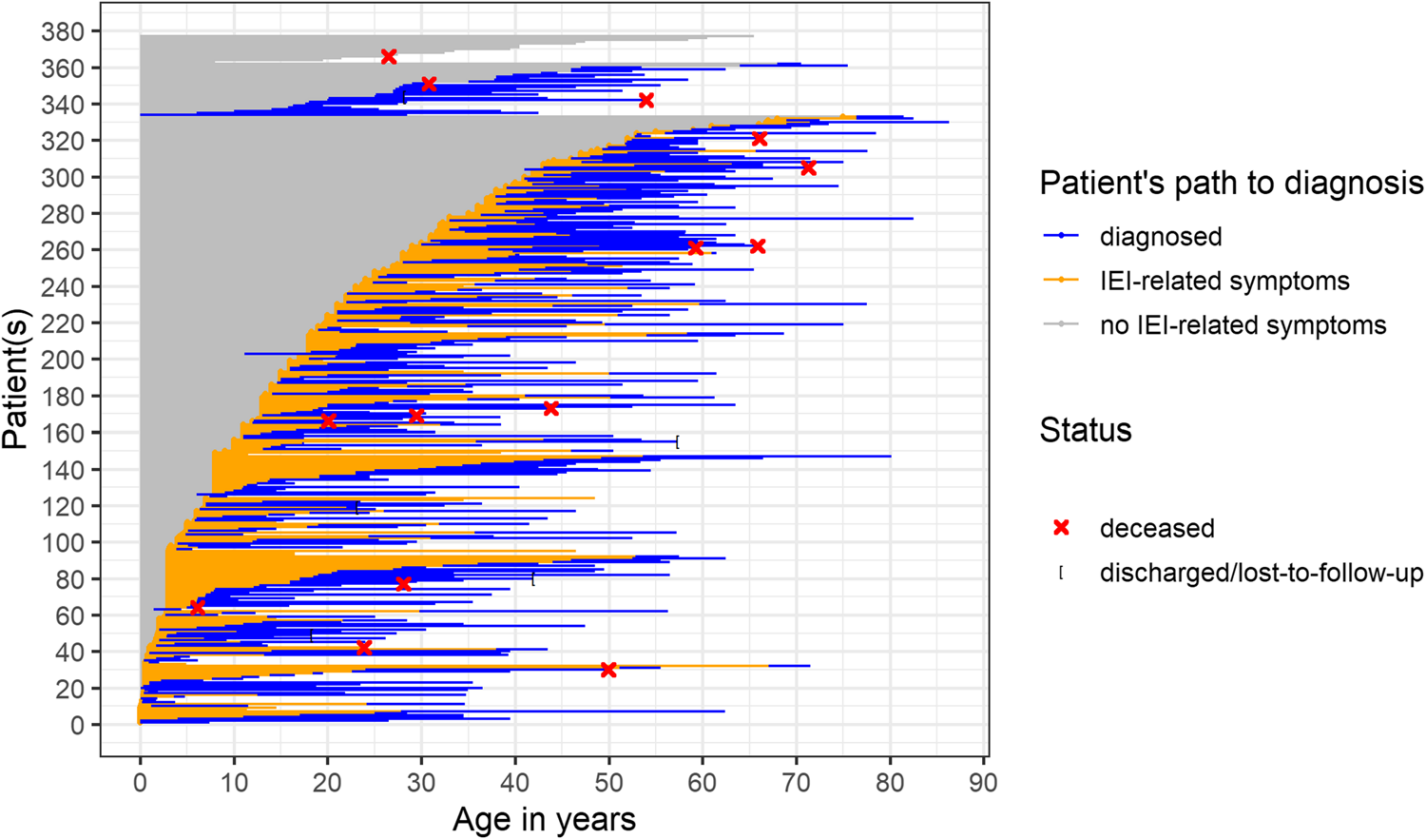
Patient’s path to diagnosis. Depicted are patients who reported symptoms related to inborn error of immunity (IEI) (*n* = 385). For patients at the top, date of symptom onset (*n* = 45) and/or date of diagnosis was unknown (*n* = 33) or they were genetically diagnosed before developing symptoms (*n* = 1). If only the partial information of the year was known, the middle of the year was used for the graph. In cases when the disease onset was given in a 5-year range, the middle of the range was used

For some genetic causes of IEI with — or at risk for — rare multi-organ autoimmunity, incomplete penetrance and varying expressivity were reported [15, 16]. Therefore, we analyzed the age at onset of the first symptom which was suggestive for an IEI. Symptom onset spanned across a wide range of ages. The median age of symptom onset was 12 years (interquartile range (IQR) 3–26.3) (Fig. 2B; Fig. 3). Though male and female patients seem to be affected equally, male patients experienced onset of symptoms significantly earlier than female patients (Fig. 2D). An X-linked mutation could only be verified for six patients, suggesting other gender- and sex-related environmental factors and/or genetic contributions. The median age at diagnosis was 25 years (IQR 13–41). The comparison of the distribution of age at onset and age at diagnosis makes clear that even patients with early onset of symptoms may experience a considerable diagnostic delay (Fig. 2B; Fig. 3).

The availability of genetic analysis has increased within the past decades and could affect the diagnostic delay over time. However, assessing this change with the current patient sample is difficult. This is due to a selection bias as most patients were included alive. Therefore, patients with a late onset of symptoms decades ago are underrepresented in our registry, as depicted in Fig. 2C by the nearly empty upper left corner of the graph. In addition, patients with a long diagnostic delay with symptom onset within the last years will still be diagnosed in the future. Therefore, our analysis of diagnostic delay can only constitute a snap shot of the patients already diagnosed.

## Discussion

We developed the GAIN registry dataset, which is able to capture detailed clinical and genetic data for these diverse diseases. To our knowledge, no other dataset was available to accomplish this goal. Previously existing data sets did not provide the possibility to describe the autoimmune manifestations and therapies in this detail and resolution of time [1, 17]. Another ongoing ESID registry advancement, the immune deficiency and dysregulation activity (IDDA) score, focuses on estimated disease burden for an overlapping patient collective, which is also relevant, but not capable of describing clinical manifestations in detail [18]. Even the data set of the autoinflammatory disease alliance registry, which was published in the end of 2022 [19], does not provide the detail needed for the description clinical manifestations in a standardized manner. This was made possible by the utilization of coding systems and ontologies, such as the ICD-11 and the HPO. The international and interlinked nature of these coding systems enables interoperability and sustainability of the GAIN dataset. The GAIN registry enables the collection of information on rare patients with inborn errors of immunity in Europe by using the European platform of the ESID registry. The GAIN registry can serve as platform to identify subgroups of patients for researchers interested in special genes or aspects of immune dysregulation. Thereby, the GAIN registry may be used in the future as a basis for etiological and diagnostic studies, and may serve as a resource to recruit patients for clinical trials on treatment options. Due to the use of broad but also detailed ontologies, other classes of diseases outside the immunological field with or without heterogeneous phenotypes could make use of the GAIN dataset. Currently, the dataset focuses on clinical manifestations of the patients and is dependent on laborious manual data entry, as most registries in Germany. To address questions of patients as “What do I have to expect from life?” other aspects such as the quality of life should complement the collection of clinical information on these rare patients. As this information is not present in standard clinical information systems, other forms of data acquisition into the registry may complement the picture in the future. The manual data entry hampers the complete and up-to-date documentation in the registry. In addition, the detailed genetic documentation needs especially trained personal. Fortunately, the Medicine Informatics Initiative in Germany takes the first steps in the right direction, by providing infrastructure for automatic data import from clinical information systems. Since physicians themselves decide whether inclusion criteria are met, and since documentation will still be mostly manual, a structure for detailed quality control is needed.

As we only have a limited number of patients (*n* = 419) at risk for or with rare multi-organ autoimmunity/autoinflammation and a limited time of follow-up for most patients, a detailed analysis of patients will follow in the upcoming years. Due to the prospective nature of the registry, the evaluation of the diagnostic delay is biased by the selection of mostly living patients, already diagnosed. Nevertheless, a 5-year (IQR 1–14) diagnostic delay of patients presenting with clinical symptoms is comparable to the diagnostic delay of 6 years (IQR 2.5–10.5) for patients with autoinflammatory disorders, collected by the UK Primary Immune Deficiency registry via the same ESID registry platform [17]. This unfortunately highlights that patients with the diverse phenotype of multi-organ autoimmunity or multi-organ autoinflammation are diagnosed later than patients with other diseases such as common variable immunodeficiencies (CVID) [17, 20].

The investigation of the total prevalence of patients with immune dysregulation syndromes is hampered by the limited number of participating centers and the inhomogeneous distribution of specialized clinical centers mainly in Germany. Therefore, the GAIN initiative tries to include centers distributed all over Europe to investigate the minimal prevalence of disorders with multi-organ autoimmunity/autoinflammation with this registry as a first step to describe and understand these rare diseases.

The GAIN registry fosters the cooperation and clinical and scientific exchange in the field of genetically determined immune dysregulation in Germany and beyond and complements the research projects of the GAIN consortium. The GAIN registry may form the basis for future research to improve patient’s life and quality of life, and helps to answer urgent questions of patients and their families.

## Supplementary Information

ESM 1

## Data Availability

All participating centers can hand in research proposals to the GAIN consortium to access data of the GAIN registry. The created data structure of the GAIN registry can be interactively explored via the publicly accessible demonstration version of the ESID registry [1].

## Abbreviations

*ABACHAI*: clinical trial investigating the safety and efficacy of the drug abatacept (ABA) in patients with CTLA4 haploinsufficiency (CHAI) or LRBA deficiency
*ATC*: Anatomical-Therapeutic-Chemical classification
*BMBF*: Bundesministerium für Bildung und Forschung, German Federal Ministry of Education and Research
*COVID-19*: corona virus disease 2019
*CVID*: common variable immunodeficiency
*DFG*: Deutsche ForschungsGemeinschaft, German Research Foundation
*ESID*: European Society for Immunodeficiencies
*et al.*: et alii, and others
*GAIN*: German multi-organ Autoimmunity Network
*GOF*: gain-of-function
*HGVS*: Human Genome Variation Society
*HPO*: Human Phenotype Ontology
*HSCT*: hematopoietic stem cell transplantation
*ICD*: International Classification of Diseases
*IEI*: inborn error of immunity
*Ig*: immunoglobulin
*IQR*: interquartile range
*IUIS*: international union of immunological societies
*n*: number; group size
*ORPHA*: standard nomenclature of rare diseases
*p*: *p*-value
*RefSeq*: Reference Sequence
*RESIST*: DFG funded excellence cluster Resolving Infection Susceptibility
*REST*: REpresentational State Transfer
*SD*: standard deviation
*WHO*: World Health Organization
