# Supplementary material for: The GAIN Registry — a New Prospective Study for Patients with Multi-organ Autoimmunity and Autoinflammation
Source: J Clin Immunol. 2023 Apr 21;43(6):1289–301. doi: 10.1007/s10875-023-01472-0 (PMC10119522; doi:10.1007/s10875-023-01472-0)
Supplement: Supplementary file 1 — ESM 1 [file 10875_2023_1472_MOESM1_ESM.pdf]

Supplementary Information

The GAIN registry – A new prospective study for patients with multi-organ autoimmunity and autoinflammation

Paulina Staus[1,2], Stephan Rusch[2], Sabine El-Helou[2,3], Gabriele Müller[2], Máté Krausz[2,4,5], Ulf Geisen[6], Andres Caballero Garcia de Oteyza[2,3], Renate Krüger[7], Shahrzad Bakhtiar[8], Min Ae Lee-Kirsch[9], Maria Fasshauer[10], Ulrich Baumann[11], Bimba Franziska Hoyer[6], João Farela Neves[12], Michael Borte[10], Maria Carrabba[13], Fabian Hauck[14], Stephan Ehl[2], Peter Bader[8], Horst von Bernuth[15,16,17], Faranaz Atschekzei[3], Mikko R. J. Seppänen[18†], Klaus Warnatz[2,4], Alexandra Nieters[2], Gerhard Kindle[2]*, Bodo Grimbacher[2,4,19,20,21]*

* These authors contributed equally to this work.

[†] On behalf of the ESID registry working party https://esid.org/Working-Parties/Registry-Working-Party

[1] Institute of Medical Biometry and Statistics, Division Methods in Clinical Epidemiology, Faculty of Medicine and Medical Center - University of Freiburg, Freiburg, Germany.

[2] Institute for Immunodeficiency, Center for Chronic Immunodeficiency (CCI), Faculty of Medicine, Medical Center - University of Freiburg, Freiburg, Germany.

[3] Department of Rheumatology and Immunology, Hannover Medical School, Hanover, Germany. Hannover Medical School, Cluster of Excellence RESIST (EXC 2155), Hanover, Germany.

[4] Department of Rheumatology and Clinical Immunology, Medical Center - University of Freiburg,

Faculty of Medicine, University of Freiburg, Freiburg, Germany.

[5] Faculty of Biology, Albert-Ludwigs-University of Freiburg, Germany.

[6] Excellence Center for Inflammation Medicine, Clinic for Rheumatology and Clinical Immunology, University Hospital Schleswig-Holstein, Campus Kiel, Kiel, Germany.

[7] Department of Pediatric Respiratory Medicine, Immunology and Critical Care Medicine, Charité - Universitätsmedizin Berlin, Berlin, Germany.

[8] Division for Stem Cell Transplantation, Immunology and Intensive Care Medicine, Hospital for Children and Adolescents, University Hospital, Goethe University, Frankfurt am Main, Germany.

[9] Department of Pediatrics, University Hospital and Medical Faculty Carl Gustav-Carus, Technische Universität Dresden, Dresden, Germany.

[10] Hospital for Children & Adolescents, St. Georg Hospital, Leipzig, Germany; Academic Teaching Hospital of the University of Leipzig, Immunodeficiency Center Leipzig (IDCL), Leipzig, Germany.

[11] Department of Paediatric Pulmonology, Allergy and Neonatology, Hannover Medical School, Hannover, Germany.

[12] Primary Immunodeficiencies Unit, Hospital Dona Estefânia, Centro Hospitalar de Lisboa Central, EPE, Lisbon, Portugal; CEDOC, Chronic Diseases Research Center, NOVA Medical School, Lisboa, Portugal.

[13] Dipartimento di Medicina Interna, Fondazione IRCCS Ca' Granda Ospedale Maggiore Policlinico, UOS Malattie Rare, Milano, Italy.

[14] Department of Pediatrics, Dr von Hauner Children's Hospital, University Hospital, Ludwig Maximilians Universität München, Munich, Germany.

[15] Berlin Institute of Health at Charité – Universitätsmedizin Berlin, Berlin, Germany.

[16] Labor Berlin GmbH, Department of Immunology, Berlin, Germany.

[17] Charité - Universitätsmedizin Berlin, corporate member of Freie Universität Berlin, Humboldt-Universität zu Berlin, and Berlin Institute of Health (BIH), Berlin-Brandenburg Center for Regenerative Therapies (BCRT), Berlin, Germany.

[18] The Rare Disease and Pediatric Research Centers, Hospital for Children and

Adolescents and Adult Immunodeficiency Unit, Inflammation Center, University of

Helsinki and HUS Helsinki, University Hospital, Helsinki, Finland.

[19] DZIF - German Center for Infection Research, Satellite Center Freiburg, Freiburg, Germany.

[20] CIBSS – Centre for Integrative Biological Signalling Studies, Albert-Ludwigs University, Freiburg, Germany.

[21] RESIST- Cluster of Excellence 2155 to Hanover Medical School, Satellite Center Freiburg, Freiburg, Germany.

Correspondence:
Prof. Dr. Bodo Grimbacher
Breisacher Str. 115, 79106 Freiburg, Germany
Tel +49 761 270-77732
Fax +49 761 270-77744
Email bodo.grimbacher@uniklinik-freiburg.de

Table S1 Centers participating in the GAIN registry and the corresponding number of patients registered

Name of the participating center	Number of registered patients
Center for Chronic Immunodeficiency, Adult Ambulance, University Hospital Freiburg; Freiburg; Germany	203

Department for Clinical Rheumatology and Immunology, Hannover Medical School; Hannover; Germany.	85
Department of Pediatric Respiratory Medicine, Immunology and Critical Care Medicine, Charité - Universitätsmedizin Berlin; Berlin; Germany	56
Centre for Children's and Youth Medicine, Division of Stem Cell Transplantation, Immunology and Intensive Care Medicine, University Hospital Frankfurt; Frankfurt; Germany	15
Center for Chronic Immunodeficiency, Pediatric Ambulance, University Hospital Freiburg; Freiburg; Germany	12
Dr. von Hauner’sches Children’s Hospital, Ludwig-Maximilians-University; Munich; Germany	10
Department of Pediatrics, University Hospital and Medical Faculty Carl Gustav-Carus, Technische Universität Dresden; Dresden; Germany	8
Centro Immunodeficienze Primitive e Sindromi Autoinfiammatorie, Department of Internal Medicine, Fondazione IRCCS Ca' Granda Ospedale Maggiore Policlinico; Milan; Italy	7
Children’s hospital St. Georg; Leipzig; Germany	7
Excellence Centre for Inflammation Medicine, Clinic for Rheumatology and Clinical Immunology, University Hospital Schleswig-Holstein, Campus Kiel; Kiel; Germany	7
Department of Pediatric Pulmonology and Neonatology, Hanover Medical School; Hannover; Germany	6
Hospital Dona Estefânia, Pediatric University Hospital, Primary Immunodeficiencies Unit; Lisbon; Portugal	3
